# High Rate of False Negative Diagnosis of Silent Patent Ductus Arteriosus on the Chest CT with 3 mm Slice-Thickness, Suggesting the Need for Analysis with Thinner Slice Thickness

**DOI:** 10.3390/tomography7030025

**Published:** 2021-07-27

**Authors:** Dongjun Lee, Minji Son, Seungmin Yoo, Sanghoon Jung, Eunju Chun, Charles S. White

**Affiliations:** 1 Department of Radiology, CHA University Bundang Medical Center, Seongnam 13497, Korea; sirious0416@chamc.co.kr (D.L.); smj3006@chamc.co.kr (M.S.); 2 Department of Radiology, Seoul National University Bundang Medical Center, Seongnam 13620, Korea; humandr@snubh.org; 3 Department of Radiology, University of Maryland, Baltimore, MD 21201, USA; cwhite@umm.edu

**Keywords:** patent ductus arteriosus, silent patent ductus arteriosus, CT

## Abstract

The purpose of this study was to evaluate the diagnostic accuracy of patent with ductus arteriosus (PDA) based on the availability of pretest information on routine chest CT with 3 mm slice-thickness. We retrospectively evaluated CT of 64 patients with PDA. The enrolled patients were categorized as group 1 (presence of pretest information) and 2 (absence of pretest information, silent PDA). CTs were read by eleven board-certified radiologists, and subsequently by two blind readers. We investigated whether a PDA was mentioned on the initial CT reading. Correct diagnosis of PDA was made in all patients with group 1 (*n* = 42). In contrast, only 13.7% were correctly diagnosed in group 2. All cases of missed PDA in group 2 were also missed by two blind readers. It is important to realize that the diagnostic accuracy of silent PDA is poor on the chest CT with 3 mm slice-thickness. Thus, use of axial CT images with the thinnest slice-thickness and multi-planar reformatted images (i.e., sagittal and coronal images) may be one way to reduce the number of missed PDA.

## 1. Introduction

Although echocardiography is the principal tool to identify patent ductus arteriosus (PDA), routine chest CT can also be a first-line imaging tool for diagnosis in adult patients with a nonspecific clinical presentation or asymptomatic PDA [1,2,3]. In clinical practice, the authors recently noted that PDA is frequently missed on chest CT with a standard slice thickness of 3 mm in patients without a known diagnosis. The diagnosis of PDA, irrespective of symptoms, is clinically important because of rare, but serious complications including endarteritis or heart failure [4]. Importantly, interventional treatment (i.e., PDA occluder insertion) can be used to prevent endarteritis or heart failure [4,5]. Thus, the aim of this study was to evaluate the differences in the diagnostic accuracy of PDA on the routine chest CT with 3 mm slice-thickness based on pretest knowledge of its presence. 

## 2. Materials and Methods

### 2.1. Study Population

**Figure 1:**
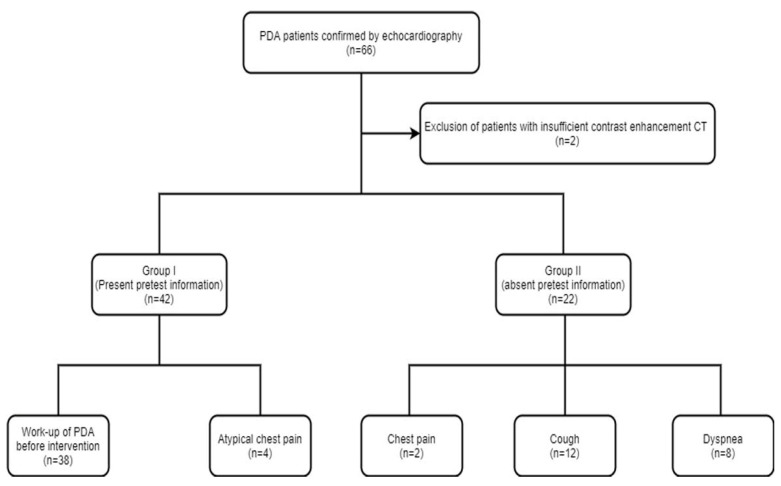
Flowchart showing the study design.

Approval was obtained from the institutional review board and informed consent was waived for this study. In a review of data at our university hospital between January 2010 and November 2019, there were 66 patients with PDA who underwent both routine chest CT and echocardiography. Two patients were excluded from this study due to insufficient enhancement (*n* = 2). Thus, a total of 64 patients with PDA (51 and 13 patients from the two institutions, respectively) comprised the study. The enrolled patients were subcategorized as group 1 (pretest information present) or group 2 (pretest information absent) based on whether pretest information regarding the diagnosis of PDA was available (Figure 1). Diagnosis of PDA was confirmed with echocardiography in all the enrolled patients.

### 2.2. Data Acquisition

Chest CT was performed in 42 patients (group 1) for the purpose of work-up of PDA prior to interventional occluder insertion (*n* = 38) and atypical chest pain (*n* = 4), respectively. In the remaining 22 patients (group 2), chest CT was performed for nonspecific chest symptoms (chest pain (*n* = 2), cough (*n* = 12), dyspnea (*n* = 8)). Chest CTs were performed on 64-slice multidetector-row CT (MDCT) (Light-speed VCT, GE HealthCare, WI, USA) or a 256-slice MDCT scanner (Brilliance iCT; Philips Medical Systems, Best, The Netherlands). Scanning parameters for CT were as follows: 100–120 kV and 400–600 mA, 3 mm slice thickness. Sixty to 100 mL of intravenous Ioversol (Optiray 320 mg/mL, Tyco Healthcare, Montreal, QC, Canada) or Iomeprol (Iomeron 400; Bracco, Milan, Italy) was injected at a flow rate of 4–6 mL/s. CT scanning was started following a 60–75 sec delay after contrast administration for chest CT.

### 2.3. Data Analysis

Chest CT studies were read by eleven board-certified radiologists (three cardiac radiologists (12–18 years of experience), seven chest radiologists (5–23 years of experience), and one cardiothoracic radiologist (18-years of experience)). We retrospectively assessed CT reports to determine if a diagnosis of PDA was mentioned at the time of CT reading. To compare CT findings of PDA between the two groups, one cardiac radiologist performed planimetry of the PDA on 3 mm curved multi-planar reformatted images; length, maximal and minimal diameter, and type of PDA were assessed. PDA was classified as one of five sub-types (type 1, cone-shaped PDA; type 2, window-shaped PDA; type 3, tubular shaped PDA; type 4, PDA with multiple constrictions; type 5 elongated shaped PDA) [6]. In particular, discrimination of type 1 (cone-shaped PDA) from type 3 PDA (tubular shaped PDA) was made by the presence or lack of difference in the diameter of PDA at the aortic and pulmonary ends. If the diameter of the aortic end of PDA was larger than that of the pulmonary end, the PDA was classified as a cone-shaped PDA (type 1 PDA). If a PDA did not fit any of the above subclassifications, it was considered an unclassified type. The diameter of the main pulmonary artery was measured on axial CT images at the level of bifurcation of the main pulmonary artery. Dilatation of the main pulmonary artery was defined as a diameter greater than 30 mm. The authors also analyzed the degree of motion artifact of the PDA (non-diagnostic motion artifact = 0, minimal or no motion artifact-diagnostic image quality = 1) with two blind readers who were unaware of study design and clinical information after the reading session. The prevalence of endarteritis was also investigated at the time of initial CT examination and during follow-up. The diagnosis of endarteritis was made according to the Duke criteria [7].

### 2.4. Diagnostic Accuracy of PDA Diagnosis by the Two Blind Readers on Chest CT

In the second part of the study, we evaluated the diagnostic accuracy of PDA (*n* = 22) without pretest information by two blind readers (5 years and 18 years of cardiothoracic experience, respectively) on chest CT. Each of the two blind readers was instructed to list all the CT findings they found. If the blind readers did not mention the diagnosis of PDA in their reading, it was considered a false negative diagnosis. 

Statistical analysis (SPSS Inc., Chicago, IL, USA) was performed using Fisher’s exact and Student’s *t*-test for categorical and continuous variables, respectively. Interobserver agreement between the two blind readers was measured using the Kappa statistic. A statistically significant difference was defined as *p* < 0.05. 

## 3. Results

Mean age of the enrolled patients was 52.5 years (range = 22–85, male/female = 15/49). There were no significant differences in the CT findings of PDA between the two groups except for mean age (Table 1 and Table 2). 

**Figure 2:**
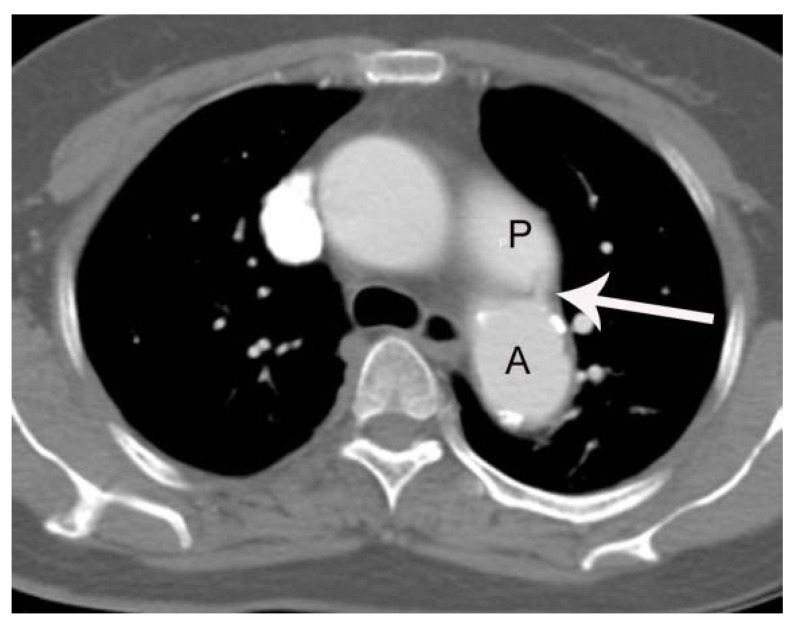
A case of PDA in a 56 -year-old female patient (group 2) missed on both the initial CT report and by the two blind readers. On axial post-contrast images with 3 mm slice-thickness at the level of tracheal carina, a small linear enhancing structure (arrow) connecting the left main pulmonary artery (P) and aortic isthmus (A) is subtly noted, but was missed on the initial CT reading.

**Figure 3:**
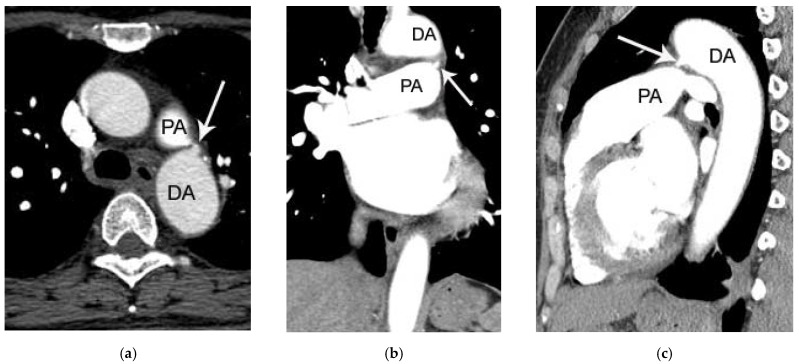
Another case of PDA in a 52-year-old female patient (group 2) missed on both initial chest CT report and by the two blind readers. On axial (**a**), coronal (**b**), and sagittal (**c**) post-contrast chest CT image, a small protruding structure (arrow) connecting the left main pulmonary artery (PA) and descending thoracic aorta (DA) is noted. Because of the small size of PDA (only 3 mm), it may be easily missed on chest CT in the absence of careful inspection.

Not surprisingly, correct diagnosis of PDA was made in all patients in group 1 (100%, 42/42). In contrast, only 3 cases (13.7%, 3/22) were correctly diagnosed in group 2 (*p* = 0.0001). The three cases with correct diagnosis in group 2 were identified by the cardiothoracic radiologist. The remaining 19 cases (Figure 2 and Figure 3) of PDA without pretest information (100% (19/19)) were missed by the seven chest radiologists (Table 3 and Table 4). Notably, 3 of the 19 patients with missed diagnosis of PDA had at least one follow-up chest CT scan (two serial chest CT scans in the two patients and four serial chest CT scans in the one patient). The diagnosis of PDA was overlooked as well on each of these serial chest CT scans; 64-slice and 256-slice MDCT was performed in 24 and 18 patients in the group 1, and 13 and 9 patients in the group 2, respectively (*p* > 0.05) (Table 5). 

All cases of PDA (*n* = 22) on chest CT without pretest information were missed by the two blind readers (Kappa value = 1.0). The image quality of all PDAs on both chest CT and cardiac CT was diagnostic based on the assessment of the two blind readers (Kappa = 1.0). On follow-up, there were two cases of endarteritis as a complication of PDA. One group 1 patient had endarteritis at initial CT examination. The other patient (group 2) had endarteritis one month after initial CT examination and died, even after intravenous administration of broad spectrum antibiotics.

## 4. Discussion

CT cannot replace echocardiography as a first-line imaging tool for the diagnosis of PDA. However, CT can be the first imaging study in patients with nonspecific chest symptoms. In these occasions, prompt CT diagnosis may prevent diagnostic delay of silent PDA [1]. In addition, CT has a better spatial resolution compared to that of echocardiography. Thus, CT may be used as a road-map to obtain anatomical details before interventional occlusion of PDA [5]. In this study, the diagnosis of PDA on CT was missed even by chest and cardiac imaging specialists in the majority of patients when there was no information pointing to the diagnosis. Several factors may account for this surprising result. First, radiologists are experiencing a heavy workload due to an increasing number of imaging studies, reducing the time to investigate every CT for a PDA. Second, anatomical characteristics of PDA on CT may play a role. Identification of relatively small diameter PDA can be difficult on axial CT images, even for chest or cardiac imaging specialists in situations where the adjacent aorta and pulmonary artery are of a large diameter and have strong post-contrast enhancement. Moreover, the missed rate in diagnosing a small PDA (2–4 mm) on a conventional chest CT scan may be higher due to the thicker slice thickness (i.e., 3 mm for chest CT) because such a small PDA is often visualized only on one or two axial CT images. Although slice-thickness used for chest CT has wide range in clinical practice (i.e., 1–5 mm), 3 mm slice-thickness is often used in order to reduce the total number of images on PACS. This fact can be problematic as most radiologists read chest CT scans primarily based on axial images, while using a coronal and sagittal view as complementary tools. In considering that there were no significant differences in the CT findings of PDA between the two groups, the high rate of false negative diagnosis may reflect a detection failure rather than an interpretation error. Third, there may be no indirect signs (i.e., CT signs of pulmonary hypertension such as enlarged main pulmonary artery, right ventricle, or right atrium) to indicate the presence of PDA other than direct visualization of PDA on CT. In fact, there were 4 cases without the main pulmonary artery enlargement in group 2 in this study. In these cases, direct identification of PDA would be the only way to arrive at a correct diagnosis. 

The high missed rate of PDA on chest CT with 3 mm slice-thickness is clinically important because detection of PDA can lead to early interventional treatment by transcatheter occluder insertion in order to prevent endarteritis or heart failure [1,2,3,4,5]. 

It is unclear what makes it possible or easier to correctly diagnose the PDA of 3 patients in the group 2 due to the small number of relevant patients. It should be mentioned that the three patients with correct diagnosis were diagnosed by one cardiothoracic radiologist who designed this study. This may be a possible reason to explain the result. However, further studies are required to address this issue.

This study has several limitations. First, it is a retrospective study with a small number of patients. Second, the comparison of diagnostic accuracy of PDA was done by only two blind readers with varying degrees of CT reading experience. Third, CT scans were performed using a 64-slice MDCT or a 256-slice MDCT scanner with a presumed variability in image quality. This factor may represent an additional source of bias. Fourth, it was not evaluated in this study whether the use of the thinnest slice-thickness supported by sagittal and coronal images may reduce the false negative diagnosis of PDA due to retrospective study design (i.e., not available sub-millimeter data on the chest CT). However, it is expected that an increase in the number of CT images demonstrating PDA on thinner slice-thickness (e.g., 1 mm) leads to the better detection of the small PDA. Fifth, an absence of ECG-gating on chest CT may have increased the prevalence of false negative diagnosis of PDA due to motion in this study. However, in general, the image quality of chest CT seems to be sufficient for the diagnosis of PDA. This may be due to a relative lack of motion artifact near the aortic arch as compared with the aortic root on chest CT. 

## 5. Conclusions

The diagnostic accuracy of silent PDA is poor on the chest CT with a 3 mm slice-thickness. Thus, use of axial CT images with the thinnest slice-thickness and multi-planar reformatted images (i.e., sagittal and coronal images) may be one way to reduce the number of missed PDA.

## Figures and Tables

**Table 1:** Clinical characteristics in groups 1 (presence of pretest information) and 2 (absence of pretest information).

	Group 1 (*n* = 42)	Group 2 (*n* = 22)	*p* Value
**Male/female**	10/32	5/17	>0.05
**Mean age**	47.9 ± 13.3 years	55.9 ± 14.2 years	0.01
**Systemic hypertension**	11/42	7/22	>0.05
**Diabetes mellitus**	4/42	5/22	>0.05
**Hemoglobin, g/dL**	13.0 ± 1.7	13.0 ± 1.9	>0.05
**Serum creatinine, mg/dL**	0.7 ± 0.1	0.8 ± 0.2	>0.05

**Table 2:** CT findings in groups 1 (presence of pretest information) and 2 (absence of pretest information).

	Group 1 (*n* = 42)	Group 2 (*n* = 22)	*p* Value
**Maximal diameter of PDA**	8.9 ± 3.4 mm	8.4 ± 3.4 mm	>0.05
**Minimal diameter of PDA**	4.5 ± 2.5 mm	4.1 ± 2.3 mm	>0.05
**Length of PDA**	8.2 ± 4.0 mm	9.8 ± 4.4 mm	>0.05
**Type of PDA**			
**Type 1**	33/42 (78.6)	16/22 (72.7)	>0.05
**Type 2**	3/42 (7.1)	1/22 (4.5)	>0.05
**Type 3**	5/42 (11.9)	4/22 (18.1)	>0.05
**Type 4**	0/42 (0)	0/22 (0)	>0.05
**Type 5**	1/42 (2.4)	0/22 (0)	>0.05
**Unclassified type**	0/42 (0)	1/22 (4.5)	>0.05
**MPA mmHg**	34.6 ± 7.8	35.4 ± 8.3	>0.05
**Pulmonary hypertension**	35/44 (79.5)	16/22 (72.7)	>0.05

PDA and MPA indicate patent ductus arteriosus and main pulmonary artery, respectively. Parenthesis indicates percentage.

**Table 3:** Clinical characteristics between the patients with correct diagnosis and those with missed diagnosis of PDA in the group 2.

	Correct Diagnosis (*n* = 3)	Missed Diagnosis (*n* = 19)
**Male/female**	1/2	7/12
**Mean age**	60.7 ± 19.1 years	56.4 ± 13.7 years
**Systemic hypertension**	1/2	6/13
**Diabetes mellitus**	1/2	5/14
**Hemoglobin, g/dL**	13.5 ± 1.5	12.2 ± 1.7
**Serum creatinine, mg/dL**	0.8 ± 0.2	0.7 ± 0.1

**Table 4:** CT findings between the patients with correct diagnosis and those with missed diagnosis of PDA in the group 2.

	Correct Diagnosis (*n* = 3)	Missed Diagnosis (*n* = 19)
**Maximal diameter of PDA**	8.6 ± 5.8 mm	8.1 ± 3.8 mm
**Minimal diameter of PDA**	4.1 ± 2.0 mm	4.0 ± 0.0 mm
**Length of PDA**	10.2 ± 3.0 mm	9.9 ± 4.7 mm
**Type of PDA**		
**Type 1**	2/3 (66.7)	13/19 (68.4)
**Type 2**	1/3 (33.3)	2/19 (10.5)
**Type 3**	0/3 (0)	1/19 (5.3)
**Type 4**	0/3 (0)	0/19 (0)
**Type 5**	0/3(0)	0/19 (0)
**Unclassified type**	0/0 (0)	1/19 (5.3)
**MPA mmHg**	31.3 ± 9.3	34.6 ± 7.4
**Pulmonary hypertension**	1/3 (33.3)	12/19 (63.2)

PDA and MPA indicate patent ductus arteriosus and main pulmonary artery, respectively. Parenthesis indicates percentage.

**Table 5:** Number of patients undertaken 64-slice MDCT and 256-slice MDCT in group 1 and 2.

	Group 1 (*n* = 42)	Group 2 (*n* = 22)	*p* Value
**64-slice MDCT**	24/42 (57.1)	13/22 (59.1)	
**256-slice MDCT**	18/42 (42.9)	9/22 (40.9)	>0.05

MDCT indicates multi-detector CT. Parenthesis indicates percentage.

## Data Availability

The data presented in this study are available on request from the corresponding author. The data are not publicly available due to privacy concerns for sharing patient data.
